# Second trimester broad ligament ectopic pregnancy: a case report

**DOI:** 10.1093/jscr/rjae084

**Published:** 2024-02-22

**Authors:** John Lugata, Onesmo Mrosso, Yusuph Mwidibo, Nasra Batchu, Bariki Mchome, Fredrick B Mbise

**Affiliations:** Department of Obstetrics and Gynecology, Kilimanjaro Christian Medical Centre, Sokoine Road, Moshi Urban Municipal, Moshi, Tanzania; Faculty of Medicine, Kilimanjaro Christian Medical University College, Sokoine Road, Moshi Urban Municipal, Moshi, Tanzania; Department of Obstetrics and Gynecology, Kilimanjaro Christian Medical Centre, Sokoine Road, Moshi Urban Municipal, Moshi, Tanzania; Faculty of Medicine, Kilimanjaro Christian Medical University College, Sokoine Road, Moshi Urban Municipal, Moshi, Tanzania; Department of Obstetrics and Gynecology, Kilimanjaro Christian Medical Centre, Sokoine Road, Moshi Urban Municipal, Moshi, Tanzania; Faculty of Medicine, Kilimanjaro Christian Medical University College, Sokoine Road, Moshi Urban Municipal, Moshi, Tanzania; Department of Obstetrics and Gynecology, Kilimanjaro Christian Medical Centre, Sokoine Road, Moshi Urban Municipal, Moshi, Tanzania; Faculty of Medicine, Kilimanjaro Christian Medical University College, Sokoine Road, Moshi Urban Municipal, Moshi, Tanzania; Department of Obstetrics and Gynecology, Kilimanjaro Christian Medical Centre, Sokoine Road, Moshi Urban Municipal, Moshi, Tanzania; Faculty of Medicine, Kilimanjaro Christian Medical University College, Sokoine Road, Moshi Urban Municipal, Moshi, Tanzania; Department of Obstetrics and Gynecology, Kilimanjaro Christian Medical Centre, Sokoine Road, Moshi Urban Municipal, Moshi, Tanzania; Faculty of Medicine, Kilimanjaro Christian Medical University College, Sokoine Road, Moshi Urban Municipal, Moshi, Tanzania

**Keywords:** ectopic pregnancy, broad ligament, emergency laparotomy, salpingectomy, low resource setting

## Abstract

An ectopic pregnancy occurs when the fertilized egg is implanted and develops outside the endometrium, i.e. in the fallopian tubes, cervix, ovary, or abdomen. It commonly presents with a history of amenorrhoea, lower abdominal pain, and slight vaginal bleeding. The fallopian tube is the most typical location for ectopic pregnancy. Two percent of reported pregnancies are ectopic pregnancy. Ectopic pregnancy remains a public health threat for women in reproductive age, and a major cause of maternal mortalities in the first trimester. In East Africa, these reports are limited, despite a great need for documentation addressing key considerations for diagnosis and management of ectopic pregnancy in these resource limited settings. In this case study, we report on 26-years-old female Gravida 5 Para 4 Living 4, who reported history of amenorrhoea for 3 months complaining of slight per vagina bleeding and lower abdominal pain for 5 days more marked at left iliac region along with generalized weakness for 2 weeks. Her vitals were stable. Pelvic ultrasound revealed empty uterus and live fetus at the left adnexa corresponding to 14 weeks 6 days with minimal free fluid in the Douglas Cul-de-sac. The patient’s final diagnosis was live extra-uterine pregnancy at 14 weeks 6 days that was managed by emergency laparotomy with salpingectomy. The patient recovered completely after surgery and was discharged in a stable condition. Ectopic pregnancy still remains one of the major causes of maternal morbidity and mortality. Early diagnosis and referral in hemodynamically state along with use of minimal access surgery or management can change the scenario of ectopic pregnancy in the developing world. Late attendance to first visit clinics is still a major concern in low resource limited settings as this could have been picked early and intervened.

**Key message:** Management of broad ligament ectopic pregnancy in the second trimester is still challenging especially in low resource settings where the clients do not attend clinics and because of unavailability of ultrasound machines to diagnose it.

## Introduction

An ectopic or extra-uterine pregnancy is one in which the blastocyst implants anywhere other than the endometrial lining of the uterine cavity. The incidence of ectopic pregnancy is 1.5%–2% of all pregnancies [1]. It is the main cause of maternal morbidity and mortality in the first trimester of pregnancies with ˃98.0% of the condition occurring in the fallopian tube, where ~70.0% of tubal pregnancy occurs at ampullar, 12.0% at isthmic, 11.0% at fimbria, and ~2.0%–3.0% are interstitial ectopic pregnancy. Age, previous infertility, smoking, previous ectopic pregnancy, history of pelvic inflammatory illnesses, any disruption of the normal architecture of the fallopian tube such as tubal ligation or reconstructive procedures, and the use of an intrauterine device (IUD) are some of the risk factors for the occurrence of ectopic pregnancies. The classic triad associated with ectopic pregnancy are delayed menses (74%), vaginal bleeding (56%), and lower abdominal pain (99%) although these symptoms are not specific and may signal other pregnancy-related conditions such as miscarriage [2, 3].

An abdominal ectopic pregnancy located at the broad ligament (within the two layers) is rare [4]. It occurs in ~1 in 300 total ectopic pregnancies and can be difficult to diagnose on imaging due to the proximity of the broad ligament to the fallopian tube [1, 5]. Often the diagnosis made by direct visualization at the time of surgery [1]. Given its rarity, there is limited literature on the management of broad ligament ectopic pregnancies. Most case reports describe patients who underwent laparotomy; however, there is an increasing number of cases treated laparoscopically [1]. Primary broad ligament ectopic pregnancy occurs within the broad ligament itself whereas secondary broad ligament pregnancy occurs following tubal rupture and grows in the broad ligament.

We present a rare case of second trimester broad ligament ectopic pregnancy at 14 weeks and 6 days that was managed by emergency ex-laparotomy.

### Case presentation

A 26-years-old female, G5P4L4, who reported history of amenorrhoea for the past 3 months with current pregnancy at 15 weeks’ gestation based on last menstrual period. She presented to the emergency unit with a complain of bleeding per vagina and abdominal pain for 5 days along with generalized weakness for 2 weeks, she also reported history of vomiting, dizziness, and awareness of heartbeats but no history of difficulty in breathing, abdominal distension, fainting, headache, or lower limb swelling. She was yet to be registered for her first antenatal visit.

She denied history of contraceptive use after her last child birth, Sexual Transmitted Disease’s, she had spontaneous conception. She belonged to poor socio-economic strata, married, and resided in a rural area. Her past medical history was unremarkable. The patient denies any history of smoking, drug use, or alcohol use prior to or during the index pregnancy.

On examination the patient had stable vitals with blood pressure of 110/95 mmHg, pulse rate 80/min, and moderately pale, abdomen was distended and tender on palpation especially on the left and right iliac and suprapubic region. The mass of 15 cm × 10 cm size was felt in abdomen. Fetal heart sounds were heard on fetal doppler. On per vaginal examination cervical, the external opening of the uterus was closed and slight bleeding was present.

**Figure 1:**
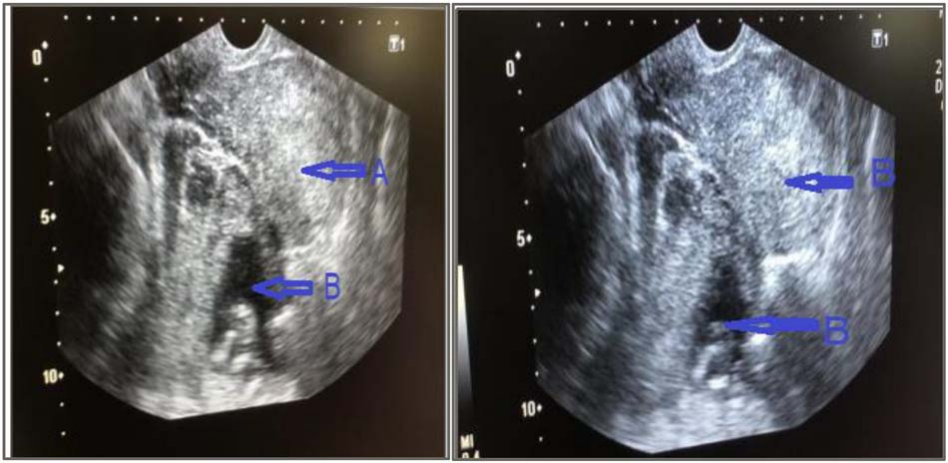
Trans vaginal ultrasound was done that showed, (a) empty uterine cavity, with no gestational sac, (b) live fetus of 14 weeks 6 days in left adnexa and moderate amount of free fluid in pelvis.

Urgent trans vaginal ultrasound was done which revealed empty uterine cavity, live fetus corresponding to 14 weeks 6 days on the left adnexa and moderate amount of free fluid in the Douglas Cul-de-sac (Fig. 1). The placenta was on the posterior aspect but its full margins were not well visualized. Impression was live intra-abdominal ectopic pregnancy at GA 14 weeks and 6 days. Her hemoglobin was 9.4 g/dl and urgent requisition for two blood units was sent. Basic laboratory parameters, including blood glucose, liver enzymes, renal function tests, and electrolytes were within normal limits The patient was taken up for emergency laparotomy after taking written and informed consent from the patient and her relative.

**Figure 2:**
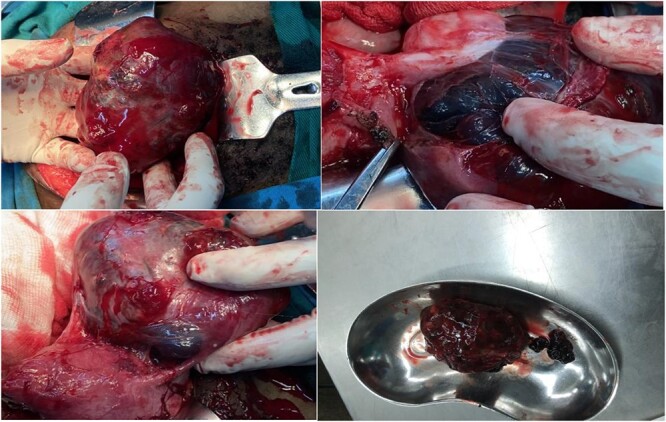
Ectopic pregnancy at the left adnexa, extending to occupy the left broad ligament, left ovary was attached on it but dissectable, size ~10.8 cm, placenta and sac together.

**Figure 3:**
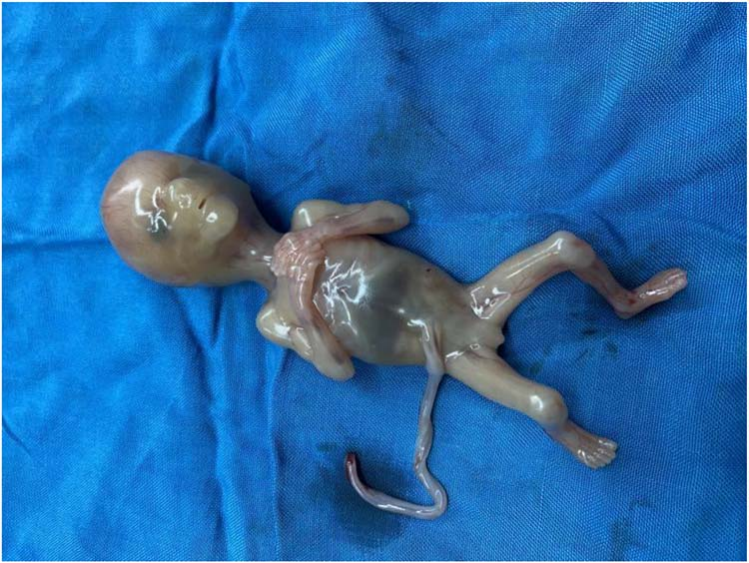
A 150 gms dead fetus of broad ligament pregnancy.

The patient was taken to the operating room (OR) without delay. She underwent an emergent exploratory laparotomy under general anesthesia and an ectopic pregnancy at the left adnexa, implanted at the broad ligament attached with left ovary and fallopian tube, and hemoperitoneum of ~50 ml was noted. Dissection was made to free the left ovary and the mass including the placenta and gestational sac and the fetus measured ~10.8 × 13 cm, (Fig. 2) was removed alongside left fallopian tube. A dead fetus of 150 gms (Fig. 3) was later extracted from the gestation sac.

The patient was kept on post-operative care including analgesics, antibiotics, intravenous fluid infusions, hematinics and she had smooth recovery and was discharged home in stable condition on day 5 after surgery to return after 2 weeks for follow-up.

## Discussion

A broad ligament ectopic pregnancy is a rare type of pregnancy that is difficult to diagnose on imaging and can result in significant morbidity due to rupture and hemoperitoneum [6]. The misdiagnosis of broad ligament ectopic pregnancies can lead to later presentations, in the second and third trimesters, and can even result in live births [7]. However, with improvement in diagnostic tools such as serum beta human chorionic gonadotropin, (β-hCG) and transvaginal sonography, ectopic pregnancies are diagnosed at much earlier gestational ages. Despite these advancements, broad ligament pregnancies are usually not diagnosed until surgery due to the lack of specific characteristics on imaging, in a recent case review of 17 broad ligament pregnancies, all but one was diagnosed intraoperatively [7]. The risk factors for broad ligament pregnancies are similar to those for tubal ectopic pregnancies and include tubal abnormalities, previous ectopic pregnancy, previous salpingectomy, pelvic infection, endometriosis, adhesions, assisted reproductive technology (ART), and presence of IUDs [7]. Although the incidence of broad ligament ectopic pregnancies is low, due to increasing use of (ART) it is likely it will rise. However, there were no apparent risk factors in our case.

Magnetic resonance imaging (MRI) provides additional information for evaluating the extent of uterine and mesenteric involvement and may help in surgical planning. Non-contrast MRI using T2 -weighted imaging is a sensitive, specific, and accurate method for evaluating ectopic pregnancy [8]. Unfortunately these advanced imaging technologies are not readily available everywhere.

The management is exploratory laparotomy. However, in a stable patient in the early gestation, laparoscopic removal can be considered for small broad ligament pregnancies [9]. In our case exploratory laparotomy was done to control the risk of bleeding. Conservative management or medical management is not recommended for broad ligament ectopic pregnancy if the diagnosis is certain. Interventional radiology also plays a crucial role to minimize morbidity and mortality by offering therapeutic options that obviate surgery and hence increases the chances of fertility preservation. Options include chemical injection of an ectopic gestational sac, uterine artery embolization, aspiration, and drainage [10].

Therefore, early diagnosis and prompt surgical intervention helps to improve the morbidity and mortality in patients with broad-ligament ectopic pregnancy.

## Conclusion

Although broad ligament pregnancy is an unusual and uncommon form of ectopic pregnancy, it should be reflected on whenever abnormal pregnancy especially with high β-hcg titers is encountered or some particular risk factors like previous surgeries and prolonged IUD usage have been accompanied. With early detection, it could be safely and effectively managed by minimally invasive methods. In conclusion what this study adds to the literature apart from describing a rare condition is that even when we are encountered with non-tubal types of ectopic pregnancy like broad ligament pregnancy, exploratory laparotomy could be safely applied in proper management of these cases especially in low setting communities.

## Data Availability

There is no data generated from this study.
